# From Algorithm to Bedside: A Clinician's Framework for AI in Practice

**DOI:** 10.1055/s-0046-1827198

**Published:** 2026-08-12

**Authors:** Alaa Abdelqader, Mohammad Alkhateeb, Abdullah Al-Marrawi, Hadi Baianonie, Fares Alahdab

**Affiliations:** 1 Alexandria University, Dentistry, Alexandria, Egypt; 2 Department of Medicine, University of Missouri, Columbia, Missouri, United States; 3 Utrecht University School of Medicine, Utrecht, The Netherlands; 4 Department of Biomedical Informatics, Biostatistics, and Medical Epidemiology, University of Missouri, Columbia, Missouri, United States; 5 Baylor College of Medicine, Houston, Texas, United States; 6 Texas McCombs School of Business, Austin, Texas, United States; 7 Department of Medicine, Division of Cardiovascular Medicine, University of Missouri, Columbia, Missouri, United States

**Keywords:** artificial intelligence, machine learning, large language model, evidence-based medicine, clinical practice

## Abstract

Artificial intelligence (AI) is entering clinical practice faster than the evidence base supporting it. Clinicians, who remain the licensed decision-makers at the bedside, increasingly find themselves as end-users of tools whose strengths, failure modes, and external validity they have had no opportunity to assess. This article offers a practical framework that does not require coding or mathematical literacy. We outline how AI is built, validated, deployed, and monitored, and where each phase typically goes wrong. We propose seven questions that clinicians can run through to evaluate any clinical AI tool in the time it takes to read an abstract, alongside a traffic-light schema for matching oversight to risk and a short list of demands clinicians should make of vendors and institutions. We then examine the deeper questions of equity, accountability, and the therapeutic relationship that AI is now forcing into view. AI literacy belongs alongside biostatistics and evidence-based medicine as a core clinical competency.

## Introduction

A nurse is paged at 2 AM because an early-warning algorithm has flagged a patient on the medical ward for possible sepsis. The patient is afebrile and clinically well, and the algorithm has now generated three sepsis alerts during the shift. The clinician must decide, in seconds, whether to trust the alert. A few floors away, a primary care physician is starting a clinic with an ambient AI scribe that listens to consultations and drafts the note. In radiology, a triage tool has just bumped a chest CT to the top of the worklist with a flag for suspected pulmonary embolism. None of these clinicians built the model. Few have seen its validation data. All of them are now responsible for what happens next.

This is the state of AI in medicine in 2026. Tools that were a research curiosity a decade ago are now embedded in the workflow, sometimes so deeply that clinicians cannot opt out. The hype cycle has predictably outpaced the evidence: systematic reviews of diagnostic deep learning have found that most studies are at high risk of bias and report performance that overstates real-world performance. 1 2 3 The recent JAMA Summit on AI emphasized that many widely adopted tools have not been rigorously evaluated for clinical effectiveness, in part because much of health AI falls outside conventional regulatory oversight. 4 At the same time, AI is producing genuine, durable advances in image interpretation, documentation, and risk stratification that no thoughtful clinician should dismiss.

The asymmetry is uncomfortable. Clinicians did not ask for most of these tools, are rarely consulted on their procurement, and yet remain the licensed professionals who must act on or override their outputs. What clinicians need is not a tutorial on machine learning. What they need is a portable framework that lets them ask whether a specific AI tool deserves their trust for a specific patient, in their setting, today, and what to demand if it does not. That is what this article aims to provide.

## AI Is Not One Thing

The term “AI” is often used as if it describes one technology. In healthcare, it does not. The most useful clinical distinction is not algorithmic but functional, and five categories now cover most of what reaches the bedside. *Diagnostic* AI tools interpret radiographs, retinal images, pathology slides, skin lesions, or electrocardiograms. *Predictive* AI tools estimate future risk of events such as sepsis, readmission, deterioration, or mortality. *Generative* AI tools, principally large language models (LLMs), produce or summarize text, including ambient scribes, draft patient messages, and triage chatbots. *Operational* AI tools support scheduling, bed management, staffing, and revenue cycle. *Patient-facing* AI tools include symptom checkers, remote-monitoring systems, and digital coaches.

These categories carry very different risks. An AI-drafted clinic note is not the same artifact as an autonomous diagnosis. A radiology worklist prioritizer is not the same as a chatbot triaging chest pain. The level of evidence, oversight, and monitoring should match the clinical stakes, not the marketing language.

Much of what is sold as AI is, mathematically, an extension of older techniques familiar to anyone who has used logistic regression. What is genuinely new is the scale of training data, the use of deep learning to extract features from raw images or free text without human-designed variables, and the emergence of foundation models whose capabilities span tasks they were not explicitly designed for. The single technical idea worth holding onto is this: AI learns patterns from training data. Everything important about a clinical AI tool, what it can do, where it fails, whom it works for, and how it may drift over time, follows from that fact. 5

## The Clinical AI Lifecycle and Where Things Go Wrong

**Fig. 1:**
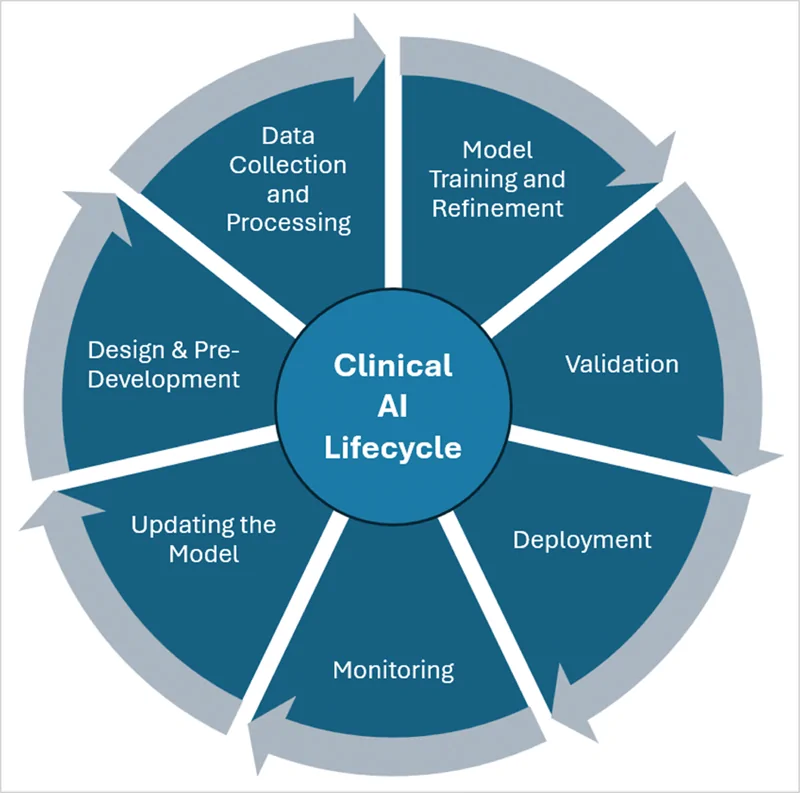
The clinical AI lifecycle and where characteristic failures may occur.

Clinical AI moves through phases relating to development, validation, deployment, and monitoring ( Fig. 1 ). Failures cluster predictably at each one.

### Development

Early decisions shape everything that follows. Who chose the training data, and does it represent the patient population that will encounter the tool? What outcome was the model asked to predict, and is that outcome a faithful representation of what we actually care about clinically? A widely cited example is an algorithm used by US health systems to allocate care-management resources. It was trained to predict future healthcare costs as a proxy for future health needs. Because Black patients with the same level of illness historically incurred lower costs, in large part due to unequal access, the algorithm systematically under-identified Black patients as high-need. 6 Mathematics worked as designed; the label was the problem.

### Validation

A model that performs well on the dataset on which it was developed tells us little about how it will perform elsewhere. Internal validation, using held-out data from the same source, almost always overstates real-world performance. External validation in patients and settings that the model has not seen is the minimum bar for clinical credibility, and even one external site does not guarantee performance at another. A living systematic review of COVID-19 prediction models examined 232 published models and rated almost all at high or unclear risk of bias. 1 A separate BMJ review of deep-learning studies claiming diagnostic parity with or superiority to clinicians found that few were prospective, fewer still were randomized, and most deviated from established reporting standards. 2 Such findings reflect a broader pattern, not a pandemic-era anomaly.

### Deployment

A model that is internally and externally valid can still fail at the bedside. The Epic Sepsis Model, deployed across hundreds of US hospitals, was found in external validation at a large academic center to have an area under the receiver-operating-characteristic curve of 0.63, to miss two-thirds of patients with sepsis, and to generate alerts on 18% of all hospitalizations. 7 Beyond raw performance, deployment introduces workflow effects: alert burden and alarm fatigue, automation bias, in which clinicians defer to the algorithm even when their own judgment would have been correct, and the slow erosion of clinical reasoning when junior clinicians outsource cognitive work to a black box. Experimental work in mammography has shown that radiologists at every level of experience can be swayed by incorrect AI suggestions, with inexperienced readers particularly vulnerable. 8

### Monitoring

Most deployed clinical AI tools are never systematically re-evaluated. Models can degrade for many reasons, including changes in coding practice, lab equipment, patient demographics, or upstream clinical practice. The COVID-19 pandemic provided a stark natural experiment in which pre-pandemic models performed unpredictably in the new environment. 5 Post-market surveillance, equity audits, and recalibration are essential but rarely funded and even more rarely mandated. An analysis of AI devices cleared by the US Food and Drug Administration found that most were authorized on the basis of retrospective single-site studies, with limited information about populations, performance, or potential failure modes. 3

A growing consensus of reporting standards exists to help authors and readers navigate these phases: TRIPOD + AI for the development and validation of clinical prediction models, 9 CONSORT-AI for randomized trials of AI interventions, 10 SPIRIT-AI for trial protocols, 11 and DECIDE-AI for early-stage clinical evaluation. 12 Familiarity with these frameworks is one of the most useful investments a clinician can make in their own AI literacy.

## Reading an AI Study Like an EBM Clinician

Reading an AI paper is, at heart, an exercise in evidence-based medicine. The questions are familiar; only the vocabulary is new. Seven questions, asked in order, will uncover most of the issues that matter ( Box 1 ).

**Box 1:** Seven questions to ask before trusting a clinical AI tool

1. **What decision does it change?** A tool without a linked action is rarely ready for practice. 2. **Were patients like mine studied?** Source population, care setting, case mix, subgroup performance. 3. **What did the model predict, and how was the label defined?** The target is half the story. 4. **What is the comparator?** Standard of care, an older score, or nothing at all. 5. **What does performance look like on outcomes that matter?** Discrimination, calibration, net benefit, prospectively, where possible. 6. **What happens when the model is wrong?** False positives and false negatives, and who pays the cost. 7. **Who is accountable when it fails?** Vendor, institution, clinician, ideally answered before deployment.

1. *What decision does this tool change* ? A vague purpose is a red flag. “Predicts deterioration” is not enough. A useful tool should make clear what clinical action it is intended to improve: bedside reassessment, escalation, additional testing, or transfer of care. If no one can explain what should happen differently when the AI output appears, the tool is not ready for practice. Risk prediction without a linked action usually becomes just another number in an already crowded electronic health record.
2. *Were patients like mine studied* ? This is the PICO question, repurposed. What was the source population? What was the care setting, the case mix, and the prevalence? A model developed and validated in tertiary referral patients may be miscalibrated in primary care, and a model trained on one health system's electronic record may behave differently in another's. 5 Performance should be reported by clinically relevant subgroups (age, sex, race and ethnicity, language, insurance status, disability, and comorbidity, site), not merely promised.
3. *What did the model predict, and how was the label defined?* Ground-truth labels in clinical AI are rarely as clean as they look. “Sepsis” can be operationalized by ICD codes, surveillance definitions, clinician documentation, or treatment patterns, and each choice yields a different model. “Pneumonia on chest X-ray” depends on which radiologist labeled the image and how. The label is the model's target; if the target is the wrong thing, even a technically excellent model will be clinically unhelpful.
4. *What is the comparator?* A model with an area under the curve of 0.85 sounds impressive until you learn that the existing risk score has an AUC of 0.83 and is free. The relevant comparator is not “nothing”; it is the current standard of care, including the clinician's own judgment, and often the right comparison is *clinician with AI support versus clinician without AI support* , rather than AI versus clinician in isolation.
5. *Performance on outcomes that matter, and prospectively?* Discrimination (the AUC or C-statistic) measures the model's ability to rank patients but says nothing about whether its probabilities are correct. Calibration, often called the Achilles heel of predictive analytics, is at least as important and is frequently neglected. 13 Beyond discrimination and calibration, the clinically meaningful question is net benefit: across the range of decision thresholds a clinician might use, does the model improve outcomes more than treating everyone or no one? Decision curve analysis was developed specifically to answer this question and is now an expected element of any serious clinical prediction paper. 14
6. *What happens when the model is wrong?* False positives and false negatives are not symmetric. A false positive in a sepsis alert costs nursing time, antibiotic exposure, and downstream alert fatigue; a false negative may cost a life. A false positive on a screening mammogram costs follow-up imaging and anxiety; a false negative delays a cancer diagnosis. A model with strong discrimination can still have a poor positive predictive value in a low-prevalence condition. A serious appraisal asks not just how often the model errs but what kinds of errors it makes, and which patients bear the cost.
7. *Who is accountable when it fails?* This is the question the AI literature is still learning to ask. Vendors disclaim responsibility in their terms of service; institutions point to vendor certifications; clinicians remain the licensed signatories on the order set. A clinician who acts on an AI recommendation, and a clinician who overrides one, are both accountable, and neither typically has full visibility into the model's logic. Asking the question in advance, ideally at procurement, is preferable to discovering the answer after an adverse event.

These seven questions cover most of what TRIPOD + AI, CONSORT-AI, SPIRIT-AI, and DECIDE-AI codify in formal detail. 9 10 11 12 A clinician who internalizes them will identify weak AI studies as quickly as they currently identify underpowered randomized trials.

## Generative AI: Useful Assistant, Unsafe Authority

Generative AI deserves its own consideration because, unlike traditional prediction models, LLMs produce language. They can summarize, translate, draft, and explain, and they can also fabricate, omit, and produce confident nonsense. The most defensible applications today are lower-risk language and administrative tasks: drafting patient instructions, simplifying medical language, preparing prior-authorization letters, summarizing long notes, and supporting patient education. Even here, human review is not optional, because a fluent paragraph can still be wrong.

Ambient AI scribes are perhaps the most rapidly adopted clinical AI tools of the past 2 years. A qualitative study of physicians in a multi-specialty pilot reported positive perspectives on cognitive demand, work-life integration, and patient engagement, with the main concerns being note length, accuracy, and editing burden. 15 A scoping review of ambient documentation systems found consistent ambulatory benefits in documentation time and clinician burnout, alongside open questions about emergency-department performance, note accuracy, and racial and dialect disparities in automatic speech recognition. 16 These are genuine, welcome gains balanced by real residual risks.

Diagnostic conversational AI is advancing quickly. Research systems such as the Articulate Medical Intelligence Explorer (AMIE) have shown strong performance in simulated diagnostic conversations 17 and in producing differential diagnoses for challenging cases, where AMIE-assisted clinicians outperformed both unassisted clinicians and clinicians with conventional search tools. 18 These are scientifically important results, but the gap between AI capability and AI-augmented human performance remains real. A randomized trial of LLM assistance in diagnostic reasoning found that physicians given access to a LLM performed no better than those using conventional resources, even though the model alone outperformed both groups. 19 The lesson is not that LLMs are useless; it is that capability in isolation does not automatically translate into improved clinician performance.

The practical rule is simple. Use generative AI as an assistant, not an authority. It can draft, organize, summarize, and challenge reasoning. It should not diagnose, prescribe, triage, or reassure patients without meaningful clinical oversight.

## Why Implementation Fails

Some AI tools fail because the model is weak. Many more fail because the implementation is bad. A tool may be technically sound but appear too late to help, be buried in the electronic health record, interrupt clinicians at the wrong time, fire so often that it becomes background noise, or identify risk without providing a recoverable action. It may shift work invisibly from physicians to nurses, pharmacists, or care managers without acknowledging the shift. It may improve a metric valued by administrators while worsening the lived experience of frontline staff.

AI is not deployed into a neutral environment. It is deployed into a clinic running behind schedule, an emergency department boarding admitted patients, a ward with staffing shortages, an inbox full of messages, and a radiology queue under pressure. Human factors are not secondary; they are part of whether the tool works. Local workflow evaluation, both before and after deployment, should map who will use the tool, when they will see it, how they will act, what competing tasks they face, and what happens to patients who do or do not receive the AI-triggered intervention. After go-live, monitoring should include alert burden, override patterns, time to action, downstream testing, treatment changes, patient outcomes, and equity, not just model performance. 4

## A Traffic-Light Framework for Clinical AI

Clinicians need a simple mental model for matching oversight to risk ( Box 2 ):

**Box 2:** A traffic-light framework for clinical AI

**Green zone (standard oversight):** patient-education drafts, plain-language rewrites, chart summaries for clinician review, administrative correspondence, and personal differential-diagnosis brainstorming.
**Yellow zone (require guardrails):** risk prediction alerts, diagnostic suggestions, imaging prioritization, treatment pathway prompts, trial matching, patient-message drafting, and medication summaries. Local validation, mandatory human review, audit trails, subgroup monitoring, and an error-reporting process.
**Red zone (avoid without formal evidence and governance):** autonomous diagnosis, autonomous prescribing, unsupervised patient triage, high-stakes decisions on unvalidated tools, deployments that transfer liability to clinicians without meaningful control.

*Green-zone* uses are lower-risk and ordinarily appropriate with standard professional review: drafting patient education materials, rewriting instructions at a lower reading level, summarizing a long chart for clinician review, preparing administrative correspondence, and helping a clinician organize a differential diagnosis for personal reflection.
*Yellow-zone* uses may be appropriate but require guardrails: risk prediction alerts, diagnostic suggestions, imaging prioritization, treatment pathway suggestions, clinical trial matching, patient messaging drafts, and medication-related summaries. Guardrails should include local validation, mandatory human review, an explicit intended use, audit trails, subgroup performance monitoring, and a process for reporting errors.
*Red-zone* uses should be avoided unless supported by formal evidence, governance, and accountability: autonomous diagnosis without clinician review, autonomous prescribing, patient triage without escalation pathways, and tools that shift liability to clinicians without giving them meaningful control. This schema is not intended to slow responsible innovation. It is intended to ensure that oversight scales with potential harm.

## Equity, Accountability, and the Therapeutic Relationship

Three structural issues deserve clinicians' sustained attention.

### Equity

Algorithmic bias is not an abstraction. The race-correction terms historically embedded in estimated glomerular filtration rate, pulmonary function testing, and obstetric risk calculators have, in many cases, systematically disadvantaged Black patients, and a critical re-examination of these practices is now underway across multiple specialties. 20 Pulse oximetry, the foundation of acute respiratory assessment, has been found to triple the rate of occult hypoxemia in Black patients compared with White patients, a finding with implications for every AI model that ingests oxygen saturation as an input. 21 Dermatology AI models trained predominantly on light skin underperform on darker skin tones, and the dermatologists labeling the training data also perform less well on those images. 22 The pattern is consistent: data inequities become algorithmic inequities, often silently.

### Accountability

When an AI tool errs, responsibility diffuses across the vendor that built it, the institution that procured it, and the clinician who acted on its output. Existing regulatory frameworks were designed for static devices, not for adaptive models that may change after deployment. In the United Kingdom and the European Union, regulators have moved toward more structured oversight of AI as a medical device, but implementation varies, and the clinician on the ward continues to carry medico-legal exposure that the device manufacturer often disclaims. Frameworks such as the NIST AI Risk Management Framework 23 and World Health Organization guidance on AI for health, including large multimodal models, 24 treat AI risk management as a continuous governance function, not a one-time technical review. The pragmatic clinical implication is that accountability cannot be delegated to the algorithm.

### The Therapeutic Relationship

Beyond technical performance, AI is altering the texture of the clinical encounter. Ambient scribes may free attention to focus on the patient, but they also risk homogenizing notes that once carried a clinician's voice, introducing hallucinations or omissions, and slowly eroding the cognitive habit of writing one's own clinical narrative. 16 Generative AI raises new questions that clinicians have not historically had to consider: what patients should be told about when AI is in the room, what consent is appropriate, and what happens to the recording afterward. These are clinical, ethical, and institutional problems that better algorithms will not solve.

### What Clinicians Should Demand

Clinicians should not be passive recipients of AI tools. From vendors and health systems, they should ask for an intended-use statement, validation data including subgroup performance, a workflow description, a description of false-positive and false-negative consequences, a monitoring plan, an equity audit plan, and a documented process for disabling or modifying the tool. For generative AI, they should also ask how outputs are grounded in source information, how hallucinations are assessed, how updates are managed, and how patient data are protected. From journals, clinicians should expect transparent reporting against TRIPOD + AI, CONSORT-AI, SPIRIT-AI, and DECIDE-AI, with clinically meaningful outcomes, not just technical metrics. From themselves and their colleagues, clinicians should expect disciplined skepticism. Skepticism is not obstruction. It is part of patient safety.

## Becoming AI-Literate

A practicing clinician in 2026 cannot opt out of AI any more than they can opt out of electronic health records. What they can do is engage with the technology on terms that protect their patients and their own professional judgment. For the next generation, AI literacy should sit alongside biostatistics and evidence-based medicine as a core clinical competency, woven into training rather than left to elective interest. For practicing clinicians, the most useful investments are pragmatic: read at least one of the TRIPOD + AI or CONSORT-AI explanatory papers; learn what discrimination, calibration, and net benefit mean and notice which is missing from the studies you read ( Box 3 ); ask vendors and institutional leaders about external validation in your patient population and plans for post-deployment monitoring; and treat AI outputs as you would a junior colleague's recommendation, with respectful scrutiny and the willingness to override when your clinical judgment disagrees. For institutional leaders, the imperative is to fund the unglamorous work of validation, equity audit, and ongoing surveillance that no vendor will do for them.

**Box 3:** Red flags in AI claims

• “Outperforms clinicians” without a realistic comparator or workflow integration • Retrospective single-site validation only • No external validation • No subgroup performance reported • No description of the intended workflow or user • No false-positive or false-negative consequence analysis • No post-deployment monitoring plan • - No clear accountability after deployment

AI in medicine is neither the salvation it is sometimes sold as nor the existential threat it is sometimes feared to be. It is a class of tools, and tools belong to those who learn to wield them well. Clinicians have done this before, with stethoscopes, with statistics, and with electronic records. Doing it again, with AI, is well within reach.
